# Midwife‐Led Ultrasound Scanning to Date Pregnancy in Malawi: Development of a Novel Training Program

**DOI:** 10.1111/jmwh.13442

**Published:** 2022-12-17

**Authors:** Alexandra Viner, Gladys Membe‐Gadama, Sonia Whyte, Doris Kayambo, Martha Masamba, Caroline J. Hollins Martin, Brian Magowan, Rebecca M. Reynolds, Sarah J Stock, Bridget Freyne, Luis Gadama

**Affiliations:** ^1^ The MRC Centre for Reproductive Health Queen's Medical Research Institute Edinburgh United Kingdom; ^2^ Obstetrics and Gynaecology Queen Elizabeth Central Hospital College of Medicine Blantyre Malawi; ^3^ Liverpool Clinical trials Centre University of Liverpool Liverpool United Kingdom; ^4^ Obstetrics and Gynaecology Mzuzu Central Hospital Mzuzu Malawi; ^5^ School of Health and Social Care Edinburgh Napier University Edinburgh United Kingdom; ^6^ Obstetrics and Gynaecology Borders General Hospital Melrose United Kingdom; ^7^ Centre for Cardiovascular Science University of Edinburgh Edinburgh United Kingdom; ^8^ Usher Institute of Population Health Sciences and Informatics University of Edinburgh Edinburgh United Kingdom; ^9^ Institute of Infection Veterinary and Ecological Sciences University of Liverpool Liverpool United Kingdom; ^10^ Obstetrics and Gynaecology Queen Elizabeth Central Hospital University of Malawi College of Medicine Blantyre Malawi

**Keywords:** Malawi, midwives, pregnancy, training, ultrasound

## Abstract

The use of ultrasound to determine gestational age is fundamental to the optimum management of pregnancy and is recommended for all women by the World Health Organization. However, this modality remains unavailable to many women in low‐income countries where trained practitioners are scarce. Although previous initiatives have demonstrated efficacy in training midwives and technicians to perform antenatal ultrasound, these programs have often been too long and too complex to be realistic within the specific constraints of this context, highlighting the need for a novel and pragmatic approach. We describe the development and piloting of a bespoke course to teach midwives 3 fundamental components of early antenatal ultrasound scanning: (1) to identify the number of fetuses, (2) to confirm fetal viability, and (3) to determine gestational age. Having established that 5 days is insufficient, we propose that the minimum duration required to train ultrasound‐naive midwives to competency is 10 days. Our completed program therefore consists of one and one‐half days of didactic teaching, followed by 8 and one‐half days of supervised hands‐on practical training in which trainees are assessed on their skills. This package has subsequently been successfully implemented across 6 sites in Malawi, where 28 midwives have achieved competency. By describing the processes involved in our cross‐continental collaboration, we explain how unexpected challenges helped shape and improve our program, demonstrating the value of preimplementation piloting and a pragmatic and adaptive approach.

## BACKGROUND

DIPLOMATIC (using eviDence, Implementation science and a clinical trial PLatform to Optimize MATernal and newborn health in low‐Income Countries) is a multidisciplinary collaboration between researchers in Malawi, Zambia, and the United Kingdom, whose aim is to reduce mortalities of children under 5 years of age through the reduction of preterm birth and stillbirth. As a fundamental component of perinatal care, the World Health Organization (WHO) has regularly cited the need for improved estimates of gestational age as a public health priority,^1^, ^2^ not only to enhance clinical care but also to strengthen the evaluation of interventions in pregnancy and the recognition and reporting of perinatal complications.

Continuing education (CE) is available for this article. To obtain CE online, please visit http://www.jmwhce.org. A CE form that includes the test questions is available in the print edition of this issue.

**QUICK POINTS**

✦ Despite the World Health Organization's recommendation that all women receive at least one ultrasound prior to 24 weeks’ gestation, this is unavailable to many women in low‐ and middle‐ income countries.
✦ We propose that ultrasound‐naive midwives can be trained to competency in basic antenatal ultrasound in 10 days.
✦ A pragmatic and context‐specific approach is essential in the development of sustainable methodology.
✦ Training programs should be evaluated iteratively prior to upscaling to maximize the chances of both immediate success and widespread adoption.
✦ The implementation of this program by local practitioners across multiple sites in Malawi is currently under evaluation.

There are a number of different ways to determine gestational age that vary in their accuracy. Early estimation using ultrasound is considered the most accurate.^3^, ^4^, ^5^ Despite the WHO recommendation that all women receive an ultrasound scan prior to 24 weeks to “estimate gestational age, improve detection of fetal anomalies and multiple pregnancies and reduce induction of labor for post term pregnancy,”^6^ ^(p xiv)^ this remains unavailable to many women living in low‐ and middle‐income countries (LMICs). Here, gestational age is derived from the last menstrual period (LMP) or by abdominal palpation, both of which are less accurate than ultrasound. Scaled provision of ultrasound is challenging for multiple reasons,^7^, ^8^, ^9^, ^10^, ^11^ with the lack of trained practitioners a recurrent barrier.^9^, ^10^

We report the development of a training package that aimed to build capacity in early pregnancy ultrasound. Several programs have demonstrated efficacy in training midwives and technicians in LMIC settings to perform antenatal ultrasound^7^, ^12^, ^13^, ^14^, ^15^, ^16^; however, they have often been difficult to sustain within existing health systems. To overcome these issues, we aimed to develop a bespoke training package that taught the fundamentals of antenatal ultrasound in the minimum time possible.

## PROGRAM DEVELOPMENT

### Approach and Rationale

The curriculum was developed by a team of obstetricians from Malawi and the United Kingdom, with input from the wider DIPLOMATIC group, including midwives, pediatricians, and social scientists. In contrast with prior initiatives^15^, ^17^, ^18^, ^19^, ^20^, ^21^ focused on delivering comprehensive training, we aimed to train ultrasound‐naive midwives to perform simple examinations, competently and independently, within the proposed training timeframe of one to 2 weeks.

As such, we required a basic curriculum containing the fundamental components of ultrasound prior to 24 weeks gestation: (1) number of fetuses, (2) confirmation of fetal viability, and (3) determination of gestational age by measurement of crown rump length (CRL) prior to 14 weeks’ gestation or fetal femur length (FL) thereafter.

Determination of gestational age after 14 weeks’ gestation may involve a combination of measurements of abdominal circumference (AC), biparietal diameter (BPD), head circumference (HC), and FL,^3^, ^4^, ^5^ although there is no universal agreement as to which combination of these performs best,^3^, ^4^, ^5^, ^22^ as the variation in the gestational ages assigned by these different approaches is minimal. Table 1 shows the accuracy of each method compared with the gold standard first trimester measurement of CRL.

**Table 1:** Degree of Agreement Between Different Ultrasound Parameters in the Second and Third Trimester and Gold Standard Crown Rump Length Measurement for the Determination of Gestational Age

	Half Width of 95% Limits of Agreement in Days		
Parameters	Ultrasound Before 24 wk^22^	Ultrasound at 24‐29+6 wk^23^	Ultrasound at 30‐36+6 wk^23^
**Combination parameters**			
Hadlock (AC/HC/FL)	±10.2	±11.1	±18.0
Intergrowth (HC/FL)	±10.6	±11.9	±19.8
**Individual parameters**			
BPD	±12.1	±14.5	±22.3
HC	±10.4	±14.6	±25.2
AC	±14.4	±16.2	±24.2
FL	±12.6	±12.8	±19.8

Abbreviations: AC, abdominal circumference; BPD, biparietal diameter; HC, head circumference; FL, femur length.

The accuracy of any ultrasound‐based parameter is dependent on the practitioner being able to obtain the measurement from a suitable ultrasound image. Unlike AC, BPD, and HC, which require the operator to obtain a circumferential measurement from a cross sectional image encompassing several specific anatomical landmarks, the FL requires only a horizontal image of the bone with both ends clearly visible, making this the easiest measurement for beginners.^4^, ^24^, ^25^ In previous programs in which health care workers in LMICs have been taught fetal biometry, FL has been the parameter performed most accurately and consistently.^20^, ^26^, ^27^ Having considered these factors, the group agreed it was both pragmatic and clinically viable to teach trainees to determine gestational age using FL from 14 weeks’ gestation.

### Content of Curriculum

Topics included an introduction to the DIPLOMATIC project, patient safety, communication, and the security and maintenance of the machines. Ultrasound specific topics included basic ultrasound physics and how to operate the machines along with scanning to establish the number of fetuses, confirmation of fetal viability, and determination of gestational age using either CRL or FL. It was also deemed important to encourage the midwives to envisage the ongoing implementation of ultrasound; therefore, topics surrounding the role of the midwife in performing antenatal ultrasound and the incorporation of scanning into routine antenatal clinics were also included.

### Development of Program and Training Materials

Having established the teaching strategy, the multinational team met 8 times via online video conferencing to co‐create a culturally appropriate and context‐specific program with supporting training materials. To maximize trainees’ practical experience, we agreed didactic teaching would form the basis of the morning sessions, with practical hands‐on scanning in the afternoons. Although it was classroom based, we sought to deliver the theoretical components interactively via a combination of presentations, small group sessions, and simulation tasks.

Every session was framed with specific learning objectives, with speaker notes and prompts provided to encourage interaction with and among the trainees. One member of the team drafted the presentation slides, before the group met online to then edit and annotate them together. A comprehensive training manual, a detailed handbook for trainees, and laminated cheat sheets were also generated using the same approach. A detailed contextualized guideline on the use of ultrasound to determine gestational age was also included.

Small group sessions focused on the role of the midwife in antenatal scanning, the incorporation of ultrasound into routine practice, and how to care for the machines. These activities were intended to encourage discussion and team problem solving, as well as to encourage trainees to take ownership of providing this service and to anticipate and troubleshoot what they envisaged may be barriers to its implementation in their facilities.

**Figure 1:**
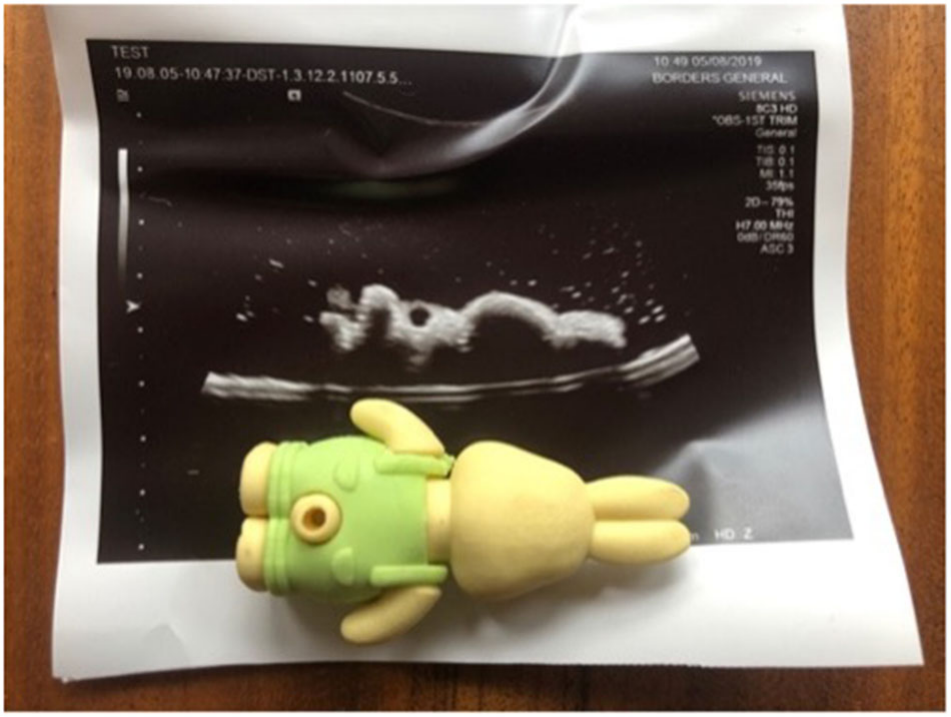
Example of Rubber Eraser and Its Appearance on Ultrasound

To help the trainees acclimatize to the machines and build confidence prior to scanning clients, we developed novel low‐cost phantoms as simulators. These were made from cheap, readily available materials that had proved robust and able to withstand the warm climate. Having trialed a variety of materials, we found that rubber erasers and chicken bones had the best echogenicity, with water as the best medium. Our phantoms therefore consisted of water filled latex gloves containing different shaped rubber erasers (Figure 1) and chicken bones in hot water bottles as FL simulators.

### Hands‐on Practical Sessions

In the afternoons, trainees were scheduled to undertake supervised ultrasound examinations on pregnant volunteers recruited during the morning from the antenatal clinics. Women provided informed written consent and, to ensure safeguarding, were scanned by a trainer first to confirm fetal viability and the absence of any fetal anomalies. Trainees then undertook structured ultrasound examinations supported by the trainers. In addition to performing the scan itself, trainees were expected to set up the machines, provide an explanation of the objectives and limitations of ultrasound, communicate the results to the pregnant volunteers, and document the results.

### Assessment

To assess trainees’ progress, their measurements of the client's fetal FL were compared with those obtained by the trainer. Using the average of 3 measurements from 3 separate images, trainees were considered competent if they demonstrated adequate global skill and their measurements fell within ±10% of that of the trainer on 5 consecutive occasions. Once achieved, remote supervision would be provided via review of their images.

### Ethical Approval

Ethical approval was obtained from the University of Malawi‐College of Medicine Research and Ethics Committee P06/19/2714. Written informed consent was acquired from both the midwives and the pregnant women, and it was made clear that it was the training program that was under evaluation, not the individual midwives.

## PROGRAM PILOT

**Figure 2:**
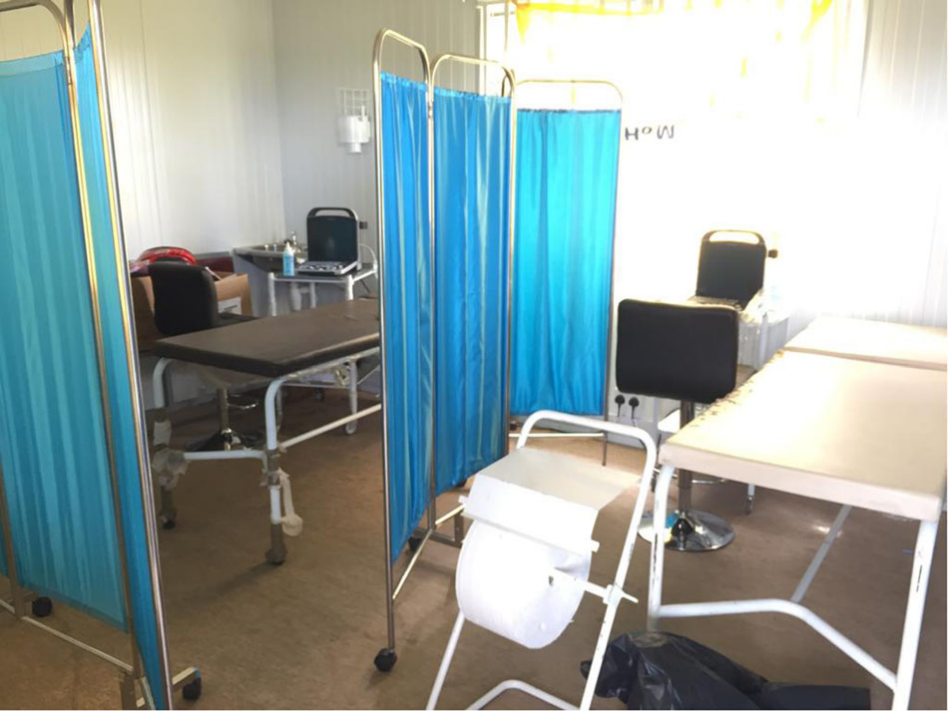
Preparing for a Hands‐on Practical Session

The materials were piloted during 2 rounds of training in Malawi in early 2020, where a total of 24 ultrasound‐naive midwives participated in 2 5‐day training courses. The first was held in Blantyre at the Queen Elizabeth Central Hospital and Ndirande Health Centre (Figure 2), with the second in Mzuzu at Mzuzu Central Hospital. Midwives were invited to participate by the Malawian obstetricians, based on their role as a key provider of antenatal care at the participating sites and in order to represent a broad range of ages and experience. The courses were conducted by the curriculum's authors in conjunction with local practitioners. The training team met at the end of each day to review which aspects of the day had been successful and what could be improved. In addition, a postcourse evaluation form surveyed the midwives’ satisfaction with the program and sought their opinion as to how the training could be enhanced, with this information helping to refine and improve the program.

### Lessons Learned from the Pilot

#### Content of the Program

Despite actively seeking clients in the first trimester, we were only able to identify 2 women who were of an appropriate gestational age to facilitate a measurement of CRL. With the majority of women in LMIC settings not attending their first antenatal visit until later in the second trimester^28^ and concerns regarding inadequate exposure to scanning clients at this gestation, we made the pragmatic decision to remove measurement of CRL from the curriculum.

Despite the simulation tasks, trainees found some aspects of the practical sessions particularly challenging, highlighting the need for some concepts to be covered in more detail prior to scanning clients. This was especially relevant to the identification of fetal parts and the manipulation of the probe to achieve the desired planes. Prior to the second iteration, we developed additional interactive sessions using plastic dolls to help trainees visualize fetal orientation and to further illustrate how manipulation of the probe would yield certain images, supplementing our preexisting materials with more examples of how fetal parts appear on ultrasound.

#### Organization of the Program

Having adapted the content of the program we also amended the schedule, moving all didactic sessions to the first 2 days and enabling the rest of the time to be spent on intensive hands‐on training. These changes proved more effective, helping trainees be better prepared for scanning clients and able to achieve far more with each interaction than previously.

#### Client Recruitment

Having anticipated that it would be easy to recruit client volunteers from antenatal clinics, we were surprised to find this challenging. By lunch time, most women had left, and of those who remained, many were unable to wait for a scan. As a result, another member of the faculty was required to recruit volunteers from different clinical areas, reducing the ratio of trainers to trainees. These issues resulted in an insufficient number of client volunteers, detracting from the trainees’ hands‐on experience and ultimately diminishing the success of the training. Prior to the second iteration, efforts were made to approach and recruit clients in advance, requesting they attend on designated days. Although not everyone attended, this did improve consistency in the availability of clients.

#### Availability of Trainers

Apart from 2 UK volunteers, all trainers participated alongside their clinical commitments, resulting in fluctuating availability depending on emergency activity. Although this had little effect on the didactic sessions, it did impact on the practical sessions in which trainees derive maximal benefit from close supervision and support.

#### Inequality of Scanning Opportunities

Throughout both iterations of the course, we noted inconsistencies in the overall number of scans performed by individual trainees. Although problems with client recruitment certainly contributed, the discrepancy also highlighted the varying degrees of confidence, engagement, and skill among the cohort. The trainees who displayed greater natural aptitude and confidence toward scanning were able to perform their examinations more quickly, with those who found it more challenging taking longer. Unfortunately, this meant that trainees progressing well had more opportunity to scan clients, often to the detriment of those who needed the additional practice. To address these inequalities, we made daily recommendations as to the number of scans expected of individual trainees, with volunteers allocated to specific groups to balance opportunity.

#### Site Used for Training

An unexpected influence on the efficacy of the training was the environment in which the hands‐on sessions were conducted. During the first iteration, these were conducted in the local health facility where the midwives were based. In contrast, practical sessions for the second iteration took place in a building adjacent to the hospital which had been purpose built for the Ebola outbreak. Although the latter seemed superior, boasting a large communal area and multiple adjoining consultation rooms, and now primarily used for training, it quickly became apparent that the midwives who underwent their training there were less engaged and less proactive than those training in their own facility, perhaps because of a relative detachment to the setting. Not only were the midwives more relaxed in their own facility, but they took greater ownership of the sessions, requiring no prompting to set up or put away materials. When complimented on their enthusiasm and commitment, they described pride in their workplace having been chosen to host the training and desire to ensure that it was efficient and successful.

#### Methods of Assessment

Although similar initiatives have been undertaken, there is no universally agreed definition of competency in performing basic antenatal ultrasound; therefore, we agreed to assess trainees based on both their global skill and the accuracy of their measurements. Without having established any specific criteria for the judgement of global skill, however, we found considerable inconsistency in how individual trainers approached this assessment and to what degree they assisted the trainees in obtaining measurements.

To standardize assessment and improve transparency we developed a formal assessment tool to facilitate observed structured clinical examinations (OSCEs). Trainees were assessed on their ability to perform specific tasks such as the optimization of their images, their communication and documentation of results, as well as 5 critical tasks aligned with the key objectives of the training. Table 2 outlines the content of the OSCE, with critical tasks noted.

**Table 2:** Content of the Observed Structured Clinical Examination

Is the trainee able to set up and turn on the scanner?
Is the trainee able to prepare and position the client appropriately?
Does the trainee ensure that they start a new examination by pressing either “end examination” or “new patient” on the scanner?
Does the trainee orientate the probe correctly?
Does the trainee assess the uterus sufficiently to establish number of fetuses?
Does the trainee determine number of fetuses correctly?^a^
Is the trainee able to identify and display the fetal heart?^a^
Based on this does the trainee correctly interpret fetal viability?^a^
Does the trainee correctly determine fetal presentation?^a^
Does the trainee optimize their images when appropriate?
Does the trainee obtain a suitable image from which to take their first measurement?
Does the trainee obtain a suitable image from which to take their second measurement?
Does the trainee obtain a suitable image from which to take their third measurement?
Is the trainee able to generate a report for their ultrasound scan?
Does the trainee consider the LMP when interpreting the scan results and correctly determine the EDB?^a^
Does the trainee document their results adequately?
Does the trainee explain their results to the client?

Abbreviations: EDB, estimated date of birth; LMP, last menstrual period.

^a^ Notes a critical task.

Source: Viner et al.^29^

For the scan to constitute a pass, trainees must perform it independently and achieve all 5 of the critical tasks and at least 6 of the remaining 12. They must also establish gestational age to within ±7 days of the trainer. Once these criteria were met for 5 consecutive scans, trainees were deemed competent to perform basic antenatal scans independently, although undoubtedly they will require, and should be provided with, ongoing support.

#### Duration of the Course

Having hypothesized that it may be possible to deliver this package over the course of a single week, it became apparent that this goal was unattainable, and trainees required more practical experience. Although most trainees developed their skills swiftly, none achieved the requirements outlined for certification within this time frame. Given the rapid progress, however, we believed that 10 days would be sufficient for the majority to train to competency. Table 3 provides a summary of recommendations for delivery of the ultrasound training package.

**Table 3:** Recommendations for Delivery of Ultrasound Training Package

Aspect of training package	Recommendation for delivery
**Organization**	Allow 10 d for the delivery of the training Complete all didactic teaching prior to commencing hands‐on sessions When possible, host hands‐on sessions in the trainee's own health facility During the practical sessions, try to maintain one trainer for every 3 trainees
**Recruitment**	Recruit client volunteers prior to the start of the training and request they attend clinic appointments on specific days Recruit sufficient volunteers to facilitate 4 scans per trainee per day
**Assessment**	Standardize assessment using the OSCE checklist

Abbreviations: OSCE, observed structured clinical examination.

### Completed Training Package

Following significant delays because of coronavirus disease 2019, this training program was delivered successfully by local practitioners across 6 sites in Malawi in early 2021. The completed program is shown in Table 4.

**Table 4:** Components of Completed Training Program

Component of training program	Specific content of sessions
**Lectures**	Introduction to ultrasound Scanning tips and orientation Introduction to the ultrasound machine How to scan for number of fetuses, fetal viability, and fetal presentation How to scan for gestational age using fetal femur length
**Small group sessions**	Incorporating ultrasound into your routine antenatal clinics Safety and storage of the ultrasound machines
**Simulation sessions**	What's in the bag? (Ice breaker) Femur length simulators Femur length: good or bad?
**Hands‐on practical sessions**	Scan practice on client volunteers directly supervised by trainers
**Formal trainee assessments**	Observed scans formally assessed by trainers

Of the participating midwives, 28 of 29 achieved competency within 10 days^29^ and have been supported to incorporate basic ultrasound into their routine practice as part of an implementation study, the results of which are pending. All were well motivated to participate, and acceptability was excellent. A more detailed evaluation of the efficacy of this program is available here.^29^

### Strengths

The strengths of this work include its pragmatic and adaptive approach, which facilitated evolution of the program via real time feedback provided by participants during each iteration. By undertaking 2 rounds of piloting, we have been able to identify and overcome a number of challenges and develop an effective and context‐specific program that is culturally appropriate and has been refined to meet the needs of those participating.

### Limitations

Although detailing the challenges of developing the program, this does not address its feasibility or the challenges of its implementation, both of which are currently being evaluated.

## CONCLUSION

We have demonstrated that it is possible to teach the basics of perinatal ultrasound in a relatively short time, creating the opportunity to upscale ultrasound skills and services in low resource settings. Although challenges were encountered, it is clear these can be overcome with adaptive processes, maximizing both the chances of immediate success and the likelihood of widespread adoption and implementation.

## CONFLICT OF INTEREST

The authors have no conflicts of interest to disclose.
